# The Health Effect of Psychostimulants: A Literature Review

**DOI:** 10.3390/ph3072333

**Published:** 2010-07-22

**Authors:** Thierry Favrod-Coune, Barbara Broers

**Affiliations:** Division of Primary Care Medicine, Geneva University Hospitals 4, Rue Gabrielle-Perret-Gentil,1211 Geneva 14, Switzerland; E-Mail: barbara.broers@hcuge.ch (B.B.)

**Keywords:** psychostimulants, cocaine, amphetamines, ecstasy (MDMA), nicotine, caffeine, khat

## Abstract

Prevalence of psychostimulant use is high, and raising in several countries. Nicotine is the legal stimulant causing the most important public health impact. Cocaine ranks among the most used illicit substances after cannabis. Stimulant medications are frequently misused. Psychostimulants can lead to addiction, have physical, psychological and social health consequences and can induce a great disease burden. The aim of the present article is to provide a literature review on the health effects of stimulants as potential drugs of abuse. It will cover essentially cocaine, amphetamines and its derivatives (including methamphetamines and 3-4-methylenedioxymethamphetamine, ecstasy), nicotine, caffeine and khat, and touch upon the issues of prescribed substances (anti-depressants, weight control medications, attention-deficit hyperactivity disorder medications, hypersomniac disorder). Their pharmacology, addictive potential, health consequences and treatment will be discussed. We used Medline for the literature review from 1990 to the date of this review, and mention the findings of human and animal studies (the latter only if they are of clinical relevance).

## 1. Introduction

Psychostimulants are the most used psychotropic substances over the world. A “psychostimulant” can be defined as a psychotropic substance with the capacity to stimulate the central nervous system. It causes excitation and elevated mood, as well as increased alertness and arousal. Its global effect is to speed up signals into the brain. A psychostimulant can also be negatively defined as a substance other than a depressant or a hallucinogenic substance. 

Beyond the worldwide use of caffeine and nicotine, illicit psychostimulants are more used in specific subgroups or cultures. Cocaine may be used in private parties as a mood and energy enhancer, methamphetamines (speed, ice) in raves or techno culture for the same reasons, and 3-4-methylene-dioxymethamphetamine (ecstasy), also known as the “love pill” in a wish to enter an empathic state. Recently, in the context of an always more individual and competitive society, the use of cocaine or methamphetamine in the professional context has been observed. A certain plant, khat, is used in specific societies in east-Africa for its psychostimulant properties. Psychostimulants (e.g. sibutramine) can be prescribed to lower appetite in obesity. This can be considered the pharmacologic part of the treatment of a huge epidemic of obesity (BMI > 30) which increased from 23% of the population in the period of 1988-1994 to 31% in the period 1999-2000 in the USA [1]. The other indication for prescribed psychostimulants (e.g. methylphenidate) is attention-hyperactivity disorder in children (US prevalence 8.3% in children aged 8-15 year old [2]) or adults (4.4% of adults aged 18-44 year old [3]). The very rare disorder narcolepsy (25-50 per 100,000 persons [4]) can warrant psychostimulant prescription (e.g. methylphenidate, modafinil or amphetamines) for the treatment of daytime sleepiness. Finally, the group of selective serotonin reuptake inhibitors and other amine reuptake inhibitors (noradrenalin) are widely used as anti-depressants but act more as correctors of an abnormal slow function of the central nervous system activity as stimulants in a direct sense of the term [5].

Caffeine is the most consumed socially acceptable stimulant, with approximately 90% of the population who consume it daily in the industrialized countries. Nicotine can be considered the most used legal stimulant with 25% of the population who use it daily in Western Europe, and 17.5% in the United States of Americas. For nicotine, the percentages are even higher in Eastern Europe and South America, and with a clear male predominance [6]. When we consider the illicit substances, the prevalence of consumption in the last year for the adult (15-64 year old) population is also quite impressive: in Europe it is estimated to be 1.3% of adults for cocaine, 0.8% for ecstasy, and 0.6% for amphetamines. The total prevalence for illicit psychostimulant use over the last 12 months will then be about 2.7% of the population last year [7]. The situation in the United States of Americas shows that cocaine (including crack) has been consumed in the last year by 7.5% of the young adults (18-25 year old), 2.2% for the younger (12-17 year old) and 2.5% for the older (26 year old and older). These percentages are respectively 3.7%, 1.3% and 0.3% for ecstasy, and 1.6%, 0.7% and 0.4% for methamphetamines [8]. Comparing to Europe the amount of people consuming illicit psychostimulants altogether in USA is more important, with about 4.5% of people using cocaine in the last year.

The WHO World Mental Health Survey Initiative followed over 85,000 people in 17 countries around the world. They found that life-time prevalence of cocaine use varied between 0 and 16%, the lowest being in China, Japan and Nigeria, and the highest being in the USA. Of interest is that the distribution is not even and cannot simply be explained by drug control policies, since country with the most severe illegal drug policies do not have lower levels of consumption than the ones with more liberal ones [9].

In a report of the National Department of Health [3], the prescribed stimulants used in the United States of Americas are found to be at the level of 3.5% of the young adults (18-25 year old), 2.3% for the younger (12-17 year old) and 0.6% for the older (26 year old and older).

The rationale for this review is to provide a comprehensive and synthetic review in English of all psychostimulants in a clinical perspective. To our knowledge several reviews on separate substances have been published, as well as a more global overview in French [10], but no global and recent overview is available. We focused on the pharmacology of each product, its addictive potential, its health consequences and the different treatment options usually admitted in case of problematic use.

We will present the different substances in order of decreasing prevalence of use in Western countries, independently of their legal status. The last section concerns the special issue of prescribed psychostimulants. 

## 2. Experimental Section

### 2.1. Methods

We have used the Cochrane database and Medline for a selective literature review from 1990 to the date of this review. If few articles were available we used earlier publications to complete. The terms inserted include “psychostimulants”, “cocaine” and “crack cocaine”, “amphetamines”, “methamphetamines”, “MDMA” and “ecstasy” (3-4-ethylenedioxymethamphetamine), “khat”, “nicotine”, “caffeine”, “appetite suppressant” and “weight control medication”, “attention-deficit hyperactivity disorder medication”, and finally “hypersomniac state medication”. We used the findings of human and animals studies, but only if they are of clinical relevance. 

## 3. Results and Discussion

### 3.1. Caffeine

Caffeine is a natural alkaloid found in plants, exactly 1,3,7-trimethylxanthine. Coffee and tea, the most widely consumed psychostimulants in the world, contain several chemical components that can be responsible for beneficial and adverse health effects, including caffeine and antioxidants (e.g. polyphenols, catechins and flavonoids) and other unidentified substances that activate the sympathetic system [11]. In Europe, the typical dose for a cup a coffee is 60-70 mg of caffeine, and about 35 mg for a cup of tea, while a glass of coca cola contains about 46 mg and an energy drink about 80 mg [12].

In the United States, the average consumption of caffeine is 280 mg per day, which is the equivalent of 2 cups per day. People taking four or more cups are considered as heavy coffee users. Coffee is preferred to tea in most developed countries (except in England and Ireland) where 71.5% of the worldwide coffee consumption [13] is used. Tea is preferred to coffee in the developing countries, particularly in Argentina, Chile, Paraguay and Uruguay, and in Asia. These countries count for 76.6% of the tea consumption in the world. The total quantity of tea consumed is much higher than coffee, and tea is thus the second beverage consumed after water.

Caffeinated soft drinks are also emerging source of caffeine intake, especially for teenagers. The caffeine content in these drinks can vary between 50 to 500 mg per can or bottle [14]. We will not discuss here substances that can be added to caffeinated beverages and potentially change their health properties, such as cream, milk or sugar.

Caffeine is rapidly absorbed and undergoes demethylation in the liver via the enzyme cytochrome P450 1A2. Defect of this enzyme is associated with prolonged caffeine half-life [15]. The genetic polymorphism in this pathway may explain differences in outcomes of studies about caffeine and its health consequences. Once in the blood, caffeine exerts its action as a potent antagonist of central and peripheral nervous system adenosine receptors. As these act as inhibitory neurotransmitters, caffeine stimulates excitatory neurotransmitters [16]. 

Several benefits can be attributed to caffeine-containing beverages. Neuro-psychological effects of caffeine are increased alertness, energy and concentration, especially if consumers are tired or night workers [17,18]. It has been suggested in a randomized study that caffeine also improves mood and working memory [19], even if some authors have postulated that these effects would be attributable to the reversal of effects due to caffeine withdrawal [20]. 

Somatic consequences of caffeine include a proven analgesic effect for tension or migraine headache at a dose superior to 65 mg [21], even if caffeine is also a potential cause of chronic migraine and rebound headache if chronically consumed [22]. A dose-effect relationship for the protection towards Parkinson’s disease [23] has been suggested, as is an association with a reduction of risk for Alzheimer’s disease [24] (relative risk of 0.7). There might also be also a protective effect of caffeine for myocardial infarction, even if it can trigger arrhythmia [25] and coronary events [26] in very susceptible individuals, probably depending on the personal caffeine metabolism (slow metabolisers being at higher risk). Caffeine containing beverages can raise the blood pressure in non-tolerant individuals, but has no long term effect on blood pressure in daily consumers [27]. Concerning the glucose metabolism, several prospective long term studies have shown that consumption of coffee or tea is associated with an improved insulin sensitivity, also in the diabetic patient [28,29], with a clear preventive action against type 2 diabetes, in normal subjects or in those with impaired glucose tolerance [30,31]. A strong inverse dose-dependent relationship between coffee drinking and alcoholic cirrhosis has been shown in a large US cohort study (relative risk ranging from 0.6 95% CI 0.6-0.8 for 1-3 cups per day to 0.2, 0.1-0.4 for drinkers of more than 4 cups) [32]. Coffee consumption seems to reduce the incidence of gout in a dose dependent manner [33]. Physical capacities are also ameliorated by caffeine [34] with a maximal benefit at a dose of 2-3 mg/kg. High caffeine intake used to be considered as doping, with a limitation of 2 to 3 cups of coffee per day (or equivalent), but this has been abandoned (ref: www.wada-ama.org).

Considering all-cause mortality, it has been shown in large cohort studies [35] (more than 100,000 persons followed for 18 years at minimum) that all-cause mortality was reduced at a relative risk of 0.8 for men (95% CI 0.62-1.04) and 0.83 for women (95% CI 0.73-0.95). The borderline statistical insignificance for men warrants further study. Most of this mortality reduction was due to a diminished cardiovascular mortality. There would be reasons to postulate that caffeine decreases risk of cancer because of its antioxidant properties, but this never has been proven scientifically, and study results are conflicting [36].

Negative consequences of caffeine include an increased risk for osteoporosis, and possibly for bone fracture, in particular for women with low calcium intake and high caffeine intake [37,38]. A link with increased risk of anxiety symptoms has been suggested, but evidence of causality is lacking. Nevertheless, caffeine has been associated with anxiety, nervousness, irritability, insomnia and even panic attacks [39,40]. In people suffering from anxiety or stress disorders, caffeinated beverages should be discussed, and discouraged if symptoms are found to be worsened by caffeine. 

The abuse or dependence potential of caffeine has not been convincingly demonstrated [41], and there is little evidence to say that tolerance develops [42]. For these reasons, no diagnosis of caffeine dependence exists in the Diagnostic and Statistical Manual of Mental Disorders-IV (DSM-IV) [43]. Still, it is clear that a caffeine withdrawal syndrome does exist. It is benign, with the principal symptoms being headache, tiredness, decreased attentiveness and contentedness [44]. This happens to 50% of people who stop drinking coffee; the most frequent symptom is the headache. It may happen from a dose as low as 100 mg/day and develop within 12 to 24 hours after the last dose. It lasts normally 1 or 2 days, but may persist as long as 9 days. A progressive reduction of caffeine intake is recommended if abstinence is planned, but no substitution is necessary if an unplanned abstinence occurs.

Summing up: caffeine has probably more advantages than risks for health, in the general population. Only patients suffering from important or unstable coronary heart disease or those at risk for or having osteoporosis should be advised to take no caffeine or in small amounts (1 to 2 cups a day). For persons with an anxiety disorder, a less clear recommendation can be made, because of a lack of arguments for a causal relationship.

### 3.2. Nicotine

As discussed in the Introduction, the prevalence of use of nicotine is basically that of tobacco, which makes nicotine the second most used psychostimulant. It is a natural alkaloid component found in tobacco leaves. Each puff of cigarette contains about 50 micrograms of nicotine. Nicotine can be absorbed through the lung and the gastro-intestinal system, but its absorption through the oral mucosa has been shown to be the principal route of absorption for smokers who do not inhale and for smokeless tobacco users [45].

The half-life of nicotine is two hours. Approximately 80 to 90% of nicotine is metabolized by the lungs, liver and kidneys. Its principle metabolite cotinine, which has a half-life of 15 to 20 hours, is active with stimulant properties. Approximately 17% of nicotine is excreted unchanged in the urine and nicotine can be found in the milk of lactating women. The metabolism of nicotine explains a racial difference in tobacco related disease. Black people have higher nicotine intake and slower cotinine metabolism, accounting for more tobacco related diseases, even with the same daily intake of nicotine [46]. An important variability exists in nicotine metabolism through the cytochrome P450-2A6 pathway, resulting in important differences in plasma nicotine and cotinine concentration, for people taking a similar dose of nicotine. A few individuals have slow metabolism, but uncertainty exists about a possible link with susceptibility to nicotine addiction [47]. The nicotinic receptor genes polymorphism account for different nicotinic acetylcholine receptors and an important variability in addictive potential for nicotine and smoking [48]. Nicotine is a hepatic enzymatic inductor and when stopped, certain drugs can require a dosage reduction. These include acetaminophen, caffeine, imipramine, oxazepam, pentazocine, propranolol and theophylline [49]. A decrease in circulating catecholamines may warrant a decrease of dose of adrenergic antagonists such as prazosin and labetalol, or an increase in dose of adrenergic substances such as isoproterenol and phenylehrine [50]. An increase in insulin absorption also exists and may necessitate a dosage reduction to avoid hypoglycemia.

Nicotine is a potent ganglionic and central nervous stimulant. It binds to nicotinic cholinergic receptors that are located in the brain, autonomic ganglia, adrenal glands and at neuromuscular junctions [49]. Nicotine induces a complex pattern of mixed sympathetic and parasympathetic responses, but its resultant major effect is sympathetic neural stimulation. It can be central or peripheral, including a catecholamine release from the adrenal glands and a direct release from vascular nerve endings. It also enhances the release of the following neurotransmitters: epinephrine, norepinephrine, dopamine, acetylcholine, serotonin, vasopressin, glutamate, nitric oxide, calcitonin growth-related peptide and β-endorphin [51]. The highly addictive potential of nicotine has been linked to its dopaminergic properties [52], and its rapid and short action. Nicotine improves cognitive performances by improving learning, memory and attention.. These effects of nicotine have been exploited in the treatment of neurodegenerative diseases, as for example in the treatment of Alzheimer’s disease patients where nicotine has been shown to attenuate the decline of the attention deficits symptomatic of this disease [53].

When considering health effects of nicotine, most studies concern smokers, so the independent effects of nicotine and smoking cannot always be separated. In the following paragraphs we have tried to separate both effects when possible. Nicotine increases cardiovascular risks by itself, without the known negative effects of smoke. It transiently increases the blood pressure with approximately 5 to 10 mmHg [54] and the heart rate up with 10 to 15 beats per minute [55]. Despite the acute effect on the arterial pressure, habitual smokers do not have higher blood pressure than nonsmokers [56]. It may be related to the decreased body weight of nicotine users, and a vasodilator effect of cotinine [57]. The cardiovascular risk is also increased by the coronary vasoconstriction and reduced coronary blood flow [58]. From studies conducted with smokers, the effects of nicotine on the cerebral circulation are both vasoconstriction and vasodilatation [59]. The vasoconstriction is partially due to thromboxane A2 release, the vasodilatation by the local formation of nitric oxide. Another deleterious effect of nicotine is a hypercoagulable state, due to platelet activation and increased fibrinogen level [60,61]. All this has a clinical importance because thrombosis is a major factor in vascular events in smokers [62]. Cigarette smoking increases the risk of myocardial infarction and sudden death more than angina pectoris does. Nicotine seems to play a role in this pathophysiology [63]. After a coronary event, smokers who continue to smoke after thrombolysis have a very high risk of reinfarction or reocclusion [64]. The risk of continued smoking in a stented coronary lesion is less clear [65]. Another way the cardiovascular damage is increased is through the endothelial dysfunction induced by nicotine or smoke. Even without atherosclerosis smokers have a paradoxical response to acetylcholine, which appears to result from impaired release of nitric oxide (NO) by the endothelium [66]. The vascular protector roles of NO are vasodilatation, reduction in platelet aggregation, smooth muscle cell proliferation, and monocytes adhesion to the endothelium.

The lipid metabolism is influenced by nicotine in the way that is largely unknown [67], but the increased cardiovascular risk seems to be independent of this change [68]. Nicotine also contributes to the development of insulin resistance. This have been suggested in a study of 40 non-obese middle-age men, in whom long term use of nicotine-containing gums was associated with the onset of insulin resistance and hyperinsulinemia [69]. 

The cardiovascular safety of nicotine replacement for smokers willing to quit has been suggested through the Lung Health Study cohort [70]. In this study on 5,887 middle-aged smokers with chronic obstructive pulmonary disease, no difference in hospital admissions for cardiovascular events was found between smokers and those who quit smoking with nicotine replacement. Two other controlled trials of nicotine replacement also provided no evidence for an increase in coronary events in patient with coronary disease [71,72].

Although there is no definite evidence that nicotine itself could induce cancer, several studies suggest that nicotine might play a role as a carcinogen, independent of smoke. This has been supported by the fact that nicotine promotes *in vivo* the growth of cancer cells and the proliferation of endothelial cells [73]. Different studies reported that nicotine suppressed apoptosis induced by different stimuli such as chemotherapeutic agents in Non Small Cell Lung Cancer treatment [74]. This explains maybe why the efficacy of the treatment of a cancer can be diminished by nicotine. 

The symptoms of nicotine withdrawal syndrome include irritability, depressed mood, restlessness, anxiety, decreased concentration, increased hunger and eating, insomnia, and craving for tobacco [75]. Nicotine withdrawal in untreated smokers produces mood disturbances comparable in intensity to those seen in depressed outpatients [76]. The withdrawal symptoms last for several weeks in the majority of cases [77].

The treatment for nicotine addiction is principally based on psychological approaches, as for other psychostimulants. The pharmacological means to enhance rate of success concerns the anti-depressant bupropion [78] and the partial agonist α4β2 nicotinic acetylcholine receptors varenicline [79]. Clinical trials have found that varenicline is superior to bupropion to quit smoking [80], and that prolonged administration of varenicline reduces relapse in abstinent smokers for 12 weeks after initial therapy [81].

In summary, independent of risks related to smoking, nicotine itself has the potential to increase the cardiovascular risk and decrease insulin resistance. Besides the advice to avoid tobacco smoke, nicotine should be avoided after a vascular event and in diabetics. Even if there is a popular belief that it is too late to stop smoking when a cancer has been diagnosed, the importance of smoking cessation should be emphasized, to enhance the treatment efficacy. Psychological support and techniques combined with pharmacologic treatment should be offered for nicotine addiction treatment.

### 3.3. Cocaine

Cocaine is the most used single illicit psychostimulant and probably the most dangerous one. It is consumed by more than 14 million people worldwide, 0.3% of the adult population age 15 to 64 years [82]. It is less available in Africa, Asia, Eastern Europe and Oceania where its consumption is less frequent. 

Cocaine is an ester alkaloid found in leaves of the Erythroxylon Coca plant. It growed typically in the Andes Mountain in South America, where the traditional oral use of the leaves is not associated in general with any negative consequences [83]. A German student, Albert Niemann, isolated the active ingredient cocaine from the leaf in 1860. It then became a ingredient of many popular preparation as coca wine or Coca-Cola, at a dose of 0.75 mg per 28 ml. Increasing reports of adverse effects as stroke and cardiac arrest warranted its government controls and its removal from Coca-Cola in 1903. Examples of its street name are “C”, “Snow”, “Aspirin”, “Coke” or “White Lady” for the snorted form, and “Crack”, “Base”, “Supercoke”, “Rock” or “Scotty” for the smoked form.

The typical pattern of use is in “binge” [84]. Shorts and intense periods of use are separated by longer period of little use or abstinence. Situations occur where cocaine is used for an extended time until the finances are exhausted or access to the substance is interrupted. The effect of cocaine is mediated through enhancement of the monoamine neurotransmitter activity in the central and peripheral nervous system. Cocaine blocks the reuptake of dopamine, norepinephrine and to a lesser degree serotonine [85,86]. Its positive psychological effects and important abuse potential are considered to be in relation to the enhancement of brain dopamine activity, dopamine being the main neurotransmitter of the brain reward and learning system [87]. Cocaine has a unique second action: it blocks voltage-gated membrane sodium ion channels and confers its anesthetic and arrhythmic properties [88]. An important effect to be known is the potential of QT prolongation [89], which can be deleterious for some patients, e.g. those with congenital long QT, or those on methadone substitution or other medication prolonging the QT interval.

Cocaine can be found in two physical forms: a hydrochloride salt and a cocaine base rock. The first is soluble in water and can be snorted or injected, its melting point is that hot (195°) that cocaine is already destroyed if heated; the second has a low melting point (95°), making it possible to smoke. It is difficult to solve into water and cannot be injected [90]. The average purity of cocaine is 50%, and it is mixed with diluents including inert fillers resembling cocaine in appearance, or active chemicals that can be toxic (e.g. benzene, acetone, lidocaine, procainamide, ephedrine, amphetamine, caffeine or PCP) [91].

After absorption, cocaine is rapidly absorbed in the blood and taken to most body organs, including the brain, heart, kidneys, adrenal glands and liver. Its crosses the placenta [92] and appears in breast milk [93]. The onset of action is very fast for the smoked or injected form (within seconds) and a couple of minute for the snorted form. The oral form, rarely observed, would take about 20 to 30 minutes to produce effects. The action lasts typically 15 to 30 minutes for the smoked or injected form, about 1 hour for the snorted form, and 2 to 3 hours for the ingested one. Those kinetic differences are thought to explain the different addiction potential of those forms, the injected or smoked form presenting the greatest addiction potential [94]. The amount of cocaine taken has also been linked to the risk of addiction. In general, the addictive potential is high and community-based interview surveys show that up to one in six persons who use cocaine will become dependent [95]. Another study found in last year users, that dependence or abuse occurred in up to 25% of people [96]. Cocaine is metabolized by the liver mainly in inactive metabolites, the only active metabolite being norcocaine. It accounts for only 5% of the metabolites but has a liver toxicity [97]. The special situation of a new compound formed when cocaine is taken with alcohol has to be mentioned. Cocaethylene has a longer half-life and is more cardiotoxic and toxic for the liver than cocaine or alcohol alone [98]. 

The effects of cocaine are increased energy and sociability, euphoria, decreased fatigue, need for sleep and appetite [99,100]. An intense pleasurable feeling has been described as a “total body orgasm”, probably following smoked or injected cocaine. With increasing dose and duration of use, the incidence of unintended adverse effect increases. They include dysphoric mood, panic attack, paranoid reactions, impaired judgment and psychotic symptom as delusions and hallucinations. The incidence of insomnia, weight loss, paranoia or hallucination can be very high even in out of treatment cocaine users, up to 40% of them [101]. Behavioral concurrent effects can be agitation, tremor, dyskinesia and repetitive or stereotyped behaviors, as compulsive research of the substance [102]. Associated signs are tachycardia, dilated pupils, sudation and nausea. Cocaine is often considered as aphrodisiac, but under its effect, if sexual desire is possibly enhanced, erection can be impaired and ejaculation delayed or inhibited [103].

The physical harms are mainly related to cardiovascular accidents and consequences of injection and sharing equipment (e.g. blood borne viruses, abscess, endocarditis). Increased cardiac oxygen demand, vasoconstriction and platelets activation are synergic in causing acute coronary syndromes [104] or cerebral strokes [105], even in young healthy individuals. Cocaine use is found in 25% of nonfatal heart attack in persons younger than 45 years [106]. It also increases the risk of cardiac arrhythmias and sudden death [107]. The cerebrovascular bed is also concerned: a US study found a 14-fold increase in the risk of ischemic or haemorrhagic stroke among cocaine users compared with matched controls (and a fourfold increased risk for methamphetamine) [108]. Other neurologic acute events concerns seizures that can happen even at the first time of use and without prior history of seizure [109].

The withdrawal syndrome is prominently psychological as with other psychostimulants. It consists in fatigue, decreased ability to feel pleasure (anhedonia), anxiety, concentration difficulties, increased appetite, sleep and dreaming. The physical signs may include musculoskeletal pain, tremor, chills, and involuntary movements [110]. This withdrawal syndrome is not dangerous, except for a risk for suicidal ideation in the initial period (which has been termed the “crash”) [111] and some myocardial ischemia described in the first week of withdrawal [112].

The physical chronic consequences are less important than the acute ones. Cardiomyopathy and myocarditis have been described [113]. Aggravation of an underlying hypertension may occur with enhanced renal disease [114]. Chronic cocaine snoring can cause the classic nasal septum perforation, oropharyngeal ulcers and osteolytic sinusitis [115]. If smoked, it can cause a moderately decreased pulmonary diffusion capacity, the spirometric tests being unchanged. The gastrointestinal chronic consequences are mainly gastric and duodenal ulcers [115]. 

More important are the neuropsychiatric long term consequences. Cocaine has been shown to be neurotoxic for the dopamine neurons, especially in the midbrain [116]. Other neuroimaging studies found cerebral gray matter atrophy, small cerebral perfusion defects and decreased D2 dopamine receptors in the striatum [117]. The clinical significance of these abnormalities are not clear, but cognitive impairment affecting visuo-motor performance, attention, verbal memory and risk-reward decision-making has been described, lasting at least several weeks after cutting down the use of cocaine [118]. 

The treatment of the intoxication consists principally of the symptomatic treatment of the underlying process. Benzodiazepines are useful to treat agitation and cardiovascular toxicity, as can be phentolamine to alleviate the vasoconstriction and hypertension due to sympathetic activation. β-Blockers have to be avoided as they can worsen the vasoconstriction due to unbalanced α stimulation [119]. One retrospective cohort study found a benefice for β-blockers, but methodological problems does not allow real conclusion [120]. α/β-blockers (e.g. labetalol) should also be avoided because of a lack of efficacy on the vasospasm [121]. Depression persisting more than two or three weeks could warrant an antidepressant therapy [122]. 

The mainstay treatment of cocaine addiction is psychosocial, with basically no approach being proved to be superior to the other [123]. Nevertheless, treatment intensity and treatment lasting a minimum three months has been associated with better outcomes [124]. Long term abstinence rates are typically under 50%. Involvement of peer self-help groups (e.g. Cocaine Anonymous, Narcotics Anonymous) seems to improve treatment outcomes [125]. No medication is proven to be an efficient treatment for cocaine dependence nor have any been labelled for this indication, but several molecules have shown some efficacy, for example topiramate (200 mg daily) [126] and tiagabine (12 or 24 mg daily) [127]. Preliminary findings suggest that GABA-b receptor agonist baclofen [128], the antidepressant citalopram [129], and the serotonin (5-HT3) receptor antagonist odansetron may diminish cocaine use in selected patients. Some other trials suggest that substitution with long lasting oral stimulants such as amphetamine might be effective [130]. The possible additive cardiovascular toxicity if the patient relapses is a problem in this setting [131]. Finally, some interesting future solutions currently under investigation include *N*-acetylcysteine (which interferes with glutamate brain activity) [132], an anti-cocaine vaccine [133] and disulfiram, possibly acting by another mechanism than that involved in the decrease of alcohol intake (probably through dopamine β-hydroxylase inhibition) [134].

In summary, cocaine is certainly one of the most dangerous and addictive psychostimulants (along with methamphetamine). Its toxicity is poorly correlated with its dosage or route of administration [135], but its addiction potential is more important if smoked or injected. Severe acute adverse effects are primarily cardiovascular (myocardial infarction, sudden death by arrhythmia), neurologic (stroke, seizure) and psychiatric (panic attack, paranoia, suicide). The simultaneous consumption with alcohol induces the formation of cocaethylene; with QT- prolonging medications it can prolong QT interval even more and induce ventricular arrhythmias. Potential users have to be warned about the risks of cocaine in general and in particular in its smoked or injected form, or if taken with alcohol, methadone or other substances susceptible to alter cardiac conduction. 

### 3.4. Methylenedioxymethamphetamine (MDMA, ecstasy) and derivatives (MDA, MDEA, PMA)

These substances are designer drugs that are classified as psychostimulants and hallucinogens. Nevertheless, MDMA’s hallucinogenic properties are light and normally limited to visual disturbances [136]. MDMA is one of the most commonly used synthetic drugs today. Its street names include “E”, “Adam”, “Clarity”, “Stacy”, “lover speed” and “essence”. After its synthesis in 1914 as a potential appetite suppressant, it was used in psychotherapy, but abandoned because of its side-effects. In the late 1980s, it became popular as a recreational drug, and prevalence of use increased in teenagers and young adults attending “rave” dance parties, night clubs and rock concerts. The prevalence of use in the last 12 months among university students has been reported to be as high as 13 to 39% in the United States and the United Kingdom [137,138], whereas the percentage of adults who have been taking MDMA in last year seems to be between 0.8 and 3.7%. Ecstasy is mainly sold in the form of pills or tablets, but may be found in powder that can be snorted or smoked. Each tablet typically contains 50 to 200 mg of active substance and is sold for about 8 to 15 Euros (11 to 21 $US).

The chemical structure of MDMA has analogies with both mescaline and methamphetamine. The drug has central stimulant and psychedelic effects mediated by serotonin (5-HT), and to a lesser proportion by dopamine [139,140]. Serotonin is thought to play a role in mood, appetite regulation and body temperature regulation. MDMA causes serotonin release, blocks its reuptake, and blocks its synthesis. The intraneuronal 5-HT stores are depleted, and the 5-HT concentration in the synaptic cleft is increased. The effect typically begins within the first hour, and lasts for 3 to 6 hours [141]. Other chemically close substances can be found mixed with MDMA in the preparations. These include MDA (methylenedioxyamphetamine), known as “love pill” because of intense euphoria and entactogen effect, MDEA (methylenedioxyethamphetamine) a substance very similar to MDMA, and PMA (paramethoxyamphetamine), suspected to be more toxic that MDMA. In an Australian study, a high proportion of persons needing emergency room admission for ecstasy intoxication actually had PMA in their urine samples [142]. The clinical effects of MDMA include a feeling of euphoria together with relaxation, empathy, and energy [143]. Sexual desire and satisfaction are enhanced [144]. The effects may include impulsive or paranoid reactions. The sense of appetite and the need to sleep are suppressed; still the feeling of thirst can sometimes be enhanced even without a water deficit after MDMA use. This can lead to absorption of large quantities of water, leading to hyponatremia, cerebral edema and even death [145]. Arousal of the body temperature as a direct consequence of serotonin stimulation, facilitated by a hot environment and sustained physical activity (dancing), can cause hyperthermia and dehydration followed by rhabdomyolysis, acute renal failure, disseminated intravascular coagulation, liver and cerebral damages, and seizures [146,147]. Researchers in Australia did a compilation of hyperthermia case reports (n=69) from MDMA and found a clear correlation of the fatality rate and the body temperature at the arrival in the emergency room (20% for temperature < or equal to 39.5°C, 78% for temperature > 42.5°C) [148]. The temperature increase was shown to be longer if cannabis was used with MDMA and the heart rate was increased [149]. The same team searched for other acute adverse reactions to MDMA and found in total ten cases of seizures without hyperthermia, twelve cases of cerebrovascular accident, and six cases (all fatal) of cardiac events. The cardiovascular events are certainly linked to the important cardiovascular stimulation as described in a controlled study showing increased heart rate (by 28 beats per minute), systolic blood pressure (by 25 mmHg), diastolic blood pressure (by 7 mmHg) and cardiac output (by 2 liters by minute) [150]. The Australian team also found 39 cases of liver injury without hyperthermia; 11 of these required liver transplantation, and six were fatal. There seems to be no clear correlation between MDMA doses and severity of outcome [151].

In the week following use of MDMA, depression and low concentration and/or memory problems are reported by users as being relatively common [152]. The risk of development of more severe psychopathological problems exists, with 31 cases of psychiatric complications described in the medical literature comprising episodes of depression, panic attacks, “flashbacks” and delusion. This can occur even after one single take of MDMA [153], or more [154]. In 12 of these cases a history of cannabis use was reported, and in eight a family or personal history of psychiatric disorder [155].

Long term toxicity of MDMA and derivatives involve principally the brain, with several studies using cerebral imaging to prove persisting abnormalities in humans who have used even moderate quantities of MDMA [156,157]. The consequences of those images involve an impairment in short-term memory function [158,159], but their long term functional consequences remain uncertain and point the need for large scale epidemiological studies. 

The treatment consists principally of symptomatic relief of consequences of MDMA use. In acute hyperthermia, the body temperature must be promptly restored, and the sodium and fluid balance also. Dantrolene, a muscle relaxant has been used to control body temperature [160]. Benzodiazepines can be useful in case of anxiety, agitation or seizures. 

It is rare that MDMA users need treatment for the use of the substance itself. The pattern of use is mostly infrequent recreational use in specific contexts without abuse or dependence, nor any consequent physical withdrawal syndrome [161]. Nevertheless, the problem may be more severe, given that some may use MDMA more often and by injection rather than orally [162]. These groups might need interventions such as classical motivational and cognitive behavioral approach as proposed for cocaine addiction, but currently no specific recommendations for treatment of MDMA misuse exist.

With regard to prescription of selective serotonin reuptake inhibitors (SSRIs), an interesting finding is that concomitant use of SSRIs and ecstasy block the usual subjective effect of MDMA [163]. Still, the risk of serotoninergic syndrome should be assessed before this pharmaceutical approach could be widely considered.

From a harm reduction perspective users should be advised to drink isotonic beverages in adequate quantity and to control their body temperature, by regularly leaving hot dance floors and taking breaks in a cool environment. Users should avoid taking MDMA in combination with ritonavir, fluoxetine or cannabis. Special attention should be taken to inform people with psychiatric or cardiovascular problems or personal or familial history of these diseases that they should absolutely avoid any MDMA use. In summary, MDMA is not a pure psychostimulant and has some hallucinogenic properties. It seems to present a smaller addiction potential than cocaine or methamphetamine. Nevertheless, acute potentially fatal reactions are described, as well as neuropsychiatric long term consequences that need further evaluation. A very serious systematic review about the health effect of recreational ecstasy has been published in 2010 [164].

### 3.5. Methamphetamine and amphetamine

These substances have been extensively discussed in the press because of its rising consumption in the US in late 90s and currently in Asia, and because of its impressive health consequences. The prevalence of annual use has declined between 2002 and 2005 [165] to 0.5% of the US adult population, but the rate of dependence within the users seems to have doubled between 2002 and 2004, passing from 10.6 to 22.3% [166]. As discussed in the introduction, the latter prevalence of use in Europe seems to be comparable, but is much higher in some Asian countries. An especially high prevalence has been described in homosexual men and is associated with a high risk of HIV transmission [167]. 

The street names of methamphetamine are “Meth”, “Crystal Meth”, “Speed”, or “Ice”. It can be easily manufactured from widely available pseudoephedrine, with a risk of explosion and burns for careless producers [168]. The substance can be taken orally, smoked, snored, injected or inserted rectally [169]. Its effect and high addictive potential can largely be compared to cocaine, but its half life is longer and about 12 hours [170]. It produces a rapid and pleasurable rush related to the release of dopamine, norepinephrine and serotonin, followed by euphoria, heightened level of alertness, and increased energy [171]. Increased libido and enhanced sexual pleasure also occur, followed by increased sexual activity with high risk behavior [172]. Dependence was clearly related to the route of administration: two-thirds of injectors were dependent, as were 58% of smokers of the substance, lower levels of dependence were seen among intranasal (33%) and oral (22%) users [173]. Amphetamine is less potent that methamphetamine but shares basically the same properties and risk. It will be detailed in the “Prescribed substance abuse” section below.

A long list of possible acute side effects is known, although their incidence is unclear. Methamphetamine sold in the street may be contaminated with multiple byproducts, including lead and α-benzylphenylamine [174]. The main acute side effects seem to be cardiovascular (myocardial infarction, stroke). One study in an emergency department in California showed 33 admissions in two years with acute chest pain and methamphetamine positive screening test [175], and 25% of these were diagnosed with acute coronary syndrome. The other adverse events are those of the others amphetamine derivatives (hyperthermia, rhabdomyolysis, acute renal failure, seizures) [176], with fatalities described [177]. Psychiatric acute reactions include anxiety, insomnia, paranoia, and psychosis.

Chronic use of methamphetamine can cause important neurological and psychiatric problems caused by dopamine depletion (anxiety, motor slowing and psychosis), as well as serotonin depletion [178] (depression and memory loss). As physical consequences, cardiomyopathy and hepatitis have been described [170]. A very typical physical appearance (“aging effect”) has been described with chronic methamphetamine use and consists of a mix of malnutrition, severe dental decay and poor hygiene.

The treatments do not differ from those for ecstasy (please refer to this substance), even if the addictive behavior could be more prominent. Treatments are mainly supportive and centered on the underlying anomaly in acute settings, and behavioral in the long term. A particular attention should be given to suicidal ideation, as the withdrawal from methamphetamine is associated with a particularly severe and prolonged depression, even more than the one of cocaine. A Cochrane review showed that fluoxetine might have modest benefit in reducing short-term methamphetamine craving, but is of limited interest since it did not permit a reduction in methamphetamine use [179]. In that paper, it is stated that imipramine can help in maintaining people in therapy. Another promising substance could be bupropion who permitted a decreased subjective methamphetamine-induced effect and craving, but in laboratory setting [180]. 

### 3.6. Khat

The Khat plant (*Celastraceus edulis*; *Catha edulis*) is a flowering perennial green tree or large shrub. It is primarily found in East Africa (Ethiopia, Kenya) and the Southwestern part of the Arabian Peninsula, and grows wild at altitudes between 1,500-2,000 m. above sea level, usually reaching 6–7 m in height [181].

The three main psychoactive ingredients of Khat leaves are alkaloids: *S*-(-)-cathinone (S-α-amino-propriophenone), norpseudoephedrine (cathine) and norephedrine, which are phenylpropylamines. They can be considered psychostimulants structurally similar to amphetamine and noradrenaline [182].

Khat consumers use the fresh leaves since cathinone is relatively unstable and oxidizes at room temperature. If left unrefrigerated for 48 hours, the leaves will contain only cathine. The leaves are chewed; the cathinone is absorbed through the oral mucosa. After ingestion, the maximal plasma concentrations of cathinone are attained after 1.5-3.5 hours [183]. The elimination half-life is around four hours [184]. Cathinone, like amphetamines, induces the release of catecholamines from presynaptic storage sites. 

Worldwide it has been estimated that there are over 10 million khat users [181,185]. Khat is mostly used in Somalia, Yemen, Kenya and Ethiopia in Muslim communities where alcohol use is prohibited and khat use legal. Different surveys suggest that in these countries life-time prevalence of khat use can be up to 80%, with prevalence of current use between 20 to 60%, and current daily use between 17 and 30% [181,185]. Most of the surveys focus on frequency of use only and not on the amount nor on negative consequences of khat use, so do not allow any conclusions on prevalence of problematic khat use. Factors that have been found to be related to daily khat use are: male gender, Muslim religion, smoking, higher level of education and family functioning. In fact, in many settings khat is used in groups, and can be considered a gathering of social significance. Special khat sessions can be organized at the occasion of festivities, sometimes in special rooms, with specific rituals and with social rules to be respected, so the function of khat use is often more social and a medium to exchange information than the search for the individual stimulant effect. In this sense, khat can be considered to have the social role of alcohol as known in many non-Muslim countries. Women do have their own sessions, but less frequently. Emigrants from the countries described seem to continue use of khat after emigration, with increased risk of legal problems [186,187]. 

In clinical experiments in healthy human volunteers khat use induces subjective and objective stimulant-like effects like increased energy, mental alertness and self-esteem. Subjects under effect of khat have been described to have increased respiratory rate, body temperature, diastolic and systolic blood pressure and heart rate, as well as mydriasis and dry mouth [187]. Chronic use can lead to insomnia, anorexia, dysphoria, concentration difficulties [181]. Khat-induced psychoses have been described in several case reports, mostly after “binge” use, and often disappear after a few days cessation of drug use, if necessary combined with an antipsychotic [181]. It is to be noted that most of these case reports concern emigrants from the khat using countries, who use khat in a “new” setting. This might suggest an increased psychotogenic risk if khat is taken in an unfamiliar setting, but also be due to publication bias.

Withdrawal symptoms that may occur after prolonged khat use have been described but seem to be usually mild. They consist of lethargy, mild depression, slight trembling, and possibly recurrent nightmares [188].

Other medical problems related to khat use that are described include: gastritis (maybe related to the high tannin concentration), delayed gastric emptying and constipation (related to the sympathomimetic effect of the Khat alkaloid) [181]. Effect on periodontal status is not clear, although prevalence of the presence of oral keratotic white lesions in the oral cavity, especially at the side where khat is used, seems to be increased [189]. There might be an increased risk of mouth and larynx cancer but available data are conflicting. Animal and human studies suggest that khat negatively influences semen count, mobility and quality. Women who take khat during pregnancy have an increased risk of dysmature infants, not of congenital malformations or stillbirth [190]. In breastfeeding women nor-pseudoephedrine can be found in breast-milk.

Although khat increases diastolic and systolic blood pressure, until at least one hour after ending a session, this effect seems more pronounced in non-tolerant subjects. Habitual users may develop tolerance to this sympathomimetic effects of khat. A few studies suggest an increased risk for acute myocardial infarction in the hours after khat use [181]. Other medical problems might be related to the presence of pesticides, fungi or parasites in the khat leaves [181]. With regard to treatment of khat misuse or dependence, two case reports (three cases) propose bromocriptine during the withdrawal phase (one case was treated for four weeks) [191,192]. 

In summary, khat use is an integrated and socially accepted, probably in general unproblematic, habit in a few Asian and African Muslim countries. As for other psycho-active substances, khat use covers the spectrum of beneficial to non-problematic use, problematic use and dependence, but criteria for, and data on the prevalence of these levels of use are lacking. Most epidemiological studies consider only the frequency of use and not the amount of substance, so recommendations of a safe level of use are lacking. Khat dependence is associated with increased morbidity and societal and economical costs. With regard to treatment, although bromocriptine prescription might have some interest there are no controlled trials available, and no specific treatment options can be recommended. We can expect standard motivational and psychological interventions in addiction medicine to have their place here too.

### 3.7. Prescribed substance abuse

The definition of prescribed substance abuse is the use of a recognized medication, usually by self-administration, in a manner that differs from medical, legal and social standards, usually by self-administration. This issue is of great interest because of its prevalence, especially concerning pain killers, opiates, sedatives and hypnotics. Diverted psychostimulant use seems to be less frequent. Nevertheless, when all medications are considered together, it is estimated by the Drug Enforcement Administration in the USA that the street value of diverted controlled medication rivals the annual street value of cocaine. The abuse of prescription drugs has risen globally, both in the US [193] and worldwide [194]. The wide availability of controlled medications for sale on the Internet also favors this trend [195]. The factors contributing to this rise are a misperception of pharmaceutical drugs as being safe, their relatively low cost, and wide availability [195].

This problem concerns the persons using diverted medication, but paradoxically also physicians who undertreat certain diseases because of their reluctance to prescribe medication with abuse potential. This phenomenon has been described for anxiety disorder, attention deficit/hyperactivity disorder (ADHD) and chronic pain [196]. 

There are criteria to help to differentiate prescription drug abuse and addiction from physical dependence on psychoactive drugs without addiction. The latter can occur within the context of good medical care (e.g. opioid dependence in chronic pain treatment without addiction). These five criteria are [197]:

- Intent (a medication is taken to treat a diagnosed illness);
- Consequences (the goal and effect of a treatment are to improve the quality of life);
- Control (a treatment is controlled cooperatively by the patient and his/her physician);
- Legality;
- Pattern (medical drug use is stable).

For the vast majority of patients with prescription drug abuse or addiction, the onset of their addictive problem precedes the initiation of prescribed controlled drugs [198]. This should help physicians to recognize most of the patients requiring particular attention with prescription. Prescribed psychostimulants susceptible to be abused are:

*Amphetamines* (including methylphenidate), prescribed for ADHD and daytime sleepiness in narcolepsy or sleep apnea syndrome. The adult dosage is usually about 20 mg per day, but recent studies showed efficacy for higher dosage, up to 70 mg per day [199]. In controlled clinical trials, amphetamine treatment allows clinically significant amelioration between 55 to 70% of patients for ADHD, and 65-85% for narcolepsy. Methylphenidate is an amphetamine like stimulant (a phenylethylamine) and catecholamine reuptake inhibitor. It is less efficient that amphetamine in narcolepsy (70% improvement of daytime wakefulness *versus* 80%) [200]. The safety of those treatments is not described in controlled studies and referral to the knowledge related to illicit use is necessary. 

*Methamphetamine*, prescribed in the USA, but not in Europe, for ADHD and obesity. The habitual dosage is 5 mg three times a day before meals for obesity, and up to 20 mg per day for ADHD. It has a lower security profile, but probably similar effects compared to other medical stimulants such as amphetamine, methylphenidate or modafinil, and its prescription should be carefully considered.

*Modafinil* is prescribed as an appetite suppressant or in sleep disorders, sibutramine as an appetite suppressant. They are stimulant-like drugs that increase levels of monoamines, serotonin, noradrenalin and dopamine. Many physicians prefer it over amphetamine, because it has less abuse potential and probably less cardiovascular risks. Still, long-term risks and abuse potential are not well known.

The serotonin selective reuptake inhibitors and the other antidepressants, even if able to create symptoms with sudden discontinuation [201], are not described to have an addictive potential. This class of medications does not enhance dopamine levers and does not produce brain rewards [202]. In summary, the prescribed psychostimulants are indicated principally, and maybe underused, for the treatment of hyperactivity/attention deficit disorders in children, adolescent and adults. Paradoxically, they are abused by an increasing number of people causing important individual and public health problems. A way to recognize potential abusers is to inquire about previous addictive behavior. 

## 4. Conclusions

The psychostimulants are by far the most used psychoactive and potentially addictive substances used all over the World and include different legal and illegal substances with different addictive potentials and health risks. They include substances that have essentially beneficial effects such as caffeine, but also some particularly dangerous substances, as cocaine and methamphetamine. Primary care physicians are probably the cornerstone for the detection of people using psychostimulants occasionally or chronically, often without any conscience of the danger linked to their behavior. They are also in the best position to provide a non-judgmental counseling and brief intervention. Patients who need more intense or specific intervention can be referred to substance abuse specialists. Treatment of psychostimulant addiction is mostly psycho-social, few medications are available, and treatment outcomes still need improvement. 
